# Comparison of ultrasound cycloplasty and transscleral cyclophotocoagulation for refractory glaucoma in Chinese population

**DOI:** 10.1186/s12886-020-01655-y

**Published:** 2020-09-29

**Authors:** Qiuli Yu, Ya Liang, Fangfang Ji, Zhilan Yuan

**Affiliations:** 1 Department of Ophthalmology, The First Affiliated Hospital of Nanjing Medical University, No. 300, Guangzhou Road, Nanjing, 210029 Jiangsu Province China; 2 Department of Ophthalmology, BenQ Medical Center, The Affiliated BenQ Hospital of Nanjing Medical University, No. 71, Hexi Road, Nanjing, 210019 Jiangsu Province China; 3 Present address: Department of Ophthalmology, The Second Affiliated Hospital of Nanjing Medical University, No. 121, Jiangjiayuan, Nanjing, 210011 Jiangsu Province China; 4 Department of Ophthalmology, The second affiliated hospital of Soochow University, No. 1055, Sanxiang Road, Suchow, 215000 Jiangsu Province China

**Keywords:** Ultrasound cycloplasty (UCP), Transscleral cyclophotocoagulation (TSCP), Refractory glaucoma, Intraocular pressure (IOP)

## Abstract

**Background:**

To compare the efficacy and safety of focused ultrasound cycloplasty (UCP) and transscleral cyclophotocoagulation (TSCP) in the treatment of refractory glaucoma in a Chinese population.

**Methods:**

We retrospectively compared twenty-eight eligible patients with refractory glaucoma, who were divided into the UCP group and TSCP group. Patients in these two groups underwent a corresponding procedure from June 2018 to February 2019. The intraocular pressure (IOP), visual acuity, the number of anti-glaucoma agents used and complications were reviewed and compared between groups. Proper statistical methods were selected according to comparison models under IBM SPSS 25 software.

**Results:**

After the 12-months follow-up, postoperative IOP and number of anti-glaucoma agents used in the two groups were both reduced than the baseline level, and the differences were statistically significant (*P* < 0.05). There were no significant differences in IOP, number of anti-glaucoma agents and the best-corrected visual acuity between the two groups at each follow-up time point (*P*>0.05). In terms of complications, the pain at 1 day after surgery in the UCP group was significantly milder than that in the TSCP group (*P* < 0.05). And there were no significant differences in other complications between the two groups (*P* > 0.05).

**Conclusions:**

Both UCP and TSCP are safe and effective methods for the treatment of refractory glaucoma. Nevertheless, pain is less severe after UCP.

## Background

Glaucoma is an irreversible neurological blindness disease with a rising incidence annually [1]. Its major pathogenic factor is high intraocular pressure (IOP), thus lowering IOP is the only recognized method to delay its progression [2]. According to the surgeons’ experience and the patients’ conditions, which may include IOP, visual field defect, and optic nerve damage, medical therapy, lasers therapy or surgery can be selected to reduce IOP [3]. Glaucoma with poor prognosis after conventional surgery is collectively referred to as refractory glaucoma, such as glaucoma that fails after multiple filtering operations, neovascular glaucoma, glaucoma following vitrectomy and glaucoma secondary to uveitis, etc. For such glaucoma patients, especially those with poor eye conditions, poor visual function or even without visual function, cyclodestructive procedure is often adopted to reduce IOP, as well as to relieve ocular and periocular pain. In recent years, laser diode transscleral cyclophotocoagulation (TSCP) has proved to be an effective method for the treatment of refractory glaucoma and gradually become a standard treatment for refractory glaucoma [4–6]. However, it still has serious complications, including loss of eyesight and even eyeball atrophy. Since the 1980s, ultrasound has been introduced in the treatment of glaucoma. In recent years, the development of high-intensity focused ultrasound technology has improved the accuracy of ultrasound cycloplasty (UCP) with a simplified therapeutic process [7]. The present study intends to compare the efficacy and complications between UCP and TSCP in treating refractory glaucoma, thereby providing references for clinical practice.

## Methods

### Subjects

We reviewed twenty-eight patients (28 eyes) who were diagnosed as refractory glaucoma in our outpatient department from June 2018 to February 2019, of whom 14 cases (14 eyes) underwent UCP and 14 cases (14 eyes) TSCP. Preoperative information was collected, including gender, age, best-corrected visual acuity (BCVA), IOP, the number of anti-glaucoma agents used, and type of primary disease. The indications for both procedures were similar, including (1) IOP was persistently higher than 21 mmHg (using applanation tonometer, 1 mmHg = 0.133 kPa) after the maximum usage of anti-glaucoma agents; (2) Patients voluntarily signed informed consent. Contra-indications were: (1) aged < 18 years; (2) evidence that the sclera was significantly thinned; (3) intraocular tumour or infection; (4) normal-tension glaucoma; and (5) choroidal hematoma. This study was approved by the ethics committee of the first affiliated Hospital of Nanjing Medical University (No. 2019-MD-168), and patients and their families were fully informed and signed informed consent.

### Treatment

#### Focused ultrasound cycloplasty

All treatments in both groups were performed in the operating room by the same experienced surgeon. The affected eyes were routinely disinfected. Retrobulbar anaesthesia was performed with ropivacaine, and topical anaesthetic was applied in the conjunctival sac. The machine (EyeOP1, Eye Tech Care, France) was prepared, the patient’s information was input to the system, and an appropriate size of probe (11 mm, 12 mm and 13 mm) was selected according to pre-treatment Ultrasound Biomicroscopy (UBM). The positioning cone was aligned and fixed on the ocular surface, and negative pressure was secured. The probe was put into the positioning cone, which was then filled with saline solution, then the treatment was activated by the surgeon. Patients with IOP of 21 ~ 35 mmHg were treated with 6 sectors; patients with IOP of 36 ~ 45 mmHg were treated with 8 sectors, and those with IOP of >45 mmHg were treated with 10 sectors. At the end of the procedure, Tobramycin and Dexamethasone Eye Ointment were applied. Postoperatively, Tobramycin Dexamethasone Eye Drops were used to prevent inflammation and infection. All anti-glaucoma medications were continued, but the dose was titrated according to the follow-up IOP. If necessary, analgesics were orally administrated after pain assessment.

#### Transscleral cyclophotocoagulation

After a similar disinfection and anaesthesia procedure, an eyelid speculum was placed on the affected eye. Oculight Slx semiconductor laser (IRIDEX, Mountain View, USA) with a wavelength of 810 nm was prepared. The fibre probe was placed 1.0–1.2 mm posterior to the limbus, which was directly over the ciliary processes. The pulse time was set to 2000 ms. The energy was set to 1200 mW initially, which was gradually increased until a significant blasting sound (“pop”) could be heard, then it was reduced by 100 mW to start treatment. The range of photocoagulation was adjusted according to the IOP, avoiding “3 and 9 o’clock” position. Specifically, for patients with IOP of 21 ~ 35 mmHg were treated with 20 laser shots, IOP of 36 ~ 45 mmHg were treated with 25 shots, and those with IOP>45 mmHg were treated with 30 shots. Postoperative management was similar to the UCP group according to the conditions of the patients.

#### Follow-up

Patients were regularly followed up in clinics after the operation at 1-day, 7-day, 1-month, 3-month, 6-month and 12-month. At every visit, routine ophthalmology examination was performed, IOP was measured by applanation tonometry, visual acuity was determined with the standardized chart (Snellen chart). For those who failed to see, “count finger”, “hand motion”, “light perception” and “no light perception” were noted accordingly. Anti-glaucoma agents were prescribed by doctors according to patients’ IOPs at each visit. IOP, the number of anti-glaucoma agents used, BCVA and complications were recorded each time. The pain was scored on the first day after surgery using a digital pain grading method, in which a 0–10 scale indicates from no pain to the most severe pain. A number was circled by patient himself/herself to show the subjective feeling of pain (0 refers to 0 grade: no pain, 1 ~ 4 refers to grade 1: mild pain, 5 ~ 9 refers to grade 2: moderate pain, 10 refers to grade 3: severe pain).

### Statistical analysis

Data were presented as mean ± standard deviation. Quantitative data were analyzed by t-test, count data were analyzed by chi-square test, and repeated measurement data were analyzed by analysis of variance of repeated measurement. A difference with *P* < 0.05 was considered statistically significant. Statistical analyses were performed using IBM SPSS 25 software (https://www.ibm.com/analytics/spss-statistics-software, IBM, USA).

## Results

### General information

A total of 28 patients with 28 eyes were included in this retrospective study. In UCP group consisted of 10 males and 4 females, including 4 cases of primary open-angle glaucoma, 3 cases of primary angle-closure glaucoma, 4 cases of secondary glaucoma and 3 cases of neovascular glaucoma. The TSCP group consisted of 8 males and 6 females, including 3 cases of primary open-angle glaucoma, 3 cases of primary angle-closure glaucoma, 4 cases of secondary glaucoma and 4 cases of neovascular glaucoma. Baseline IOP values were 43.36 ± 12.68 and 40.64 ± 10.97 mmHg in UCP and TSCP group. Patients were stratified according to baseline IOP level with a common criterion to receive an adapted range of treatment. There were no significant differences in terms of gender, mean age, preoperative BCVA, baseline IOP, types of glaucoma, and the number of anti-glaucoma agents used before surgery between the two groups (all *P* > 0.05) (Table 1).

**Table 1:** Comparison of preoperative baseline data between the two groups

		UCP	TSCP	t/χ^**2**^value	***P***-value
**Number of cases/ eyes**		14/14	14/14		
**Gender (M/F)**		10/4	8/6	0.622	0.430
**Age (**$\overline{\boldsymbol{x}}$**± s, years)**		62.71 ± 16.69	53.93 ± 14.32	−1.495	0.147
**Baseline IOP (**$\overline{\boldsymbol{x}}$**± s, mmHg)**		43.36 ± 12.68	40.64 ± 10.97	−0.607	0.549
**IOP Stratifi-cation**	**21 < IOP ≤ 35**	5	7	1.359	0.5069
	**35 < IOP ≤ 45**	1	2		
	**IOP > 45**	8	5		
**Number of anti-glaucoma agents (**$\overline{\boldsymbol{x}}$**± s)**		2.29 ± 0.83	2.36 ± 0.74	0.240	0.812
**Type of primary disease before surgery**	**OAG**	4	3	0.286	0.963
	**ACG**	3	3		
	**Secondary**	4	4		
	**NVG**	3	4		
**Best-corrected visual acuity before surgery**	**No LP**	5	5	4.800	0.187
	**LP**	2	3		
	**HM**	7	3		
	**CF**	0	3		

*OAG* Open angle glaucoma, *ACG* Angle closed glaucoma, *NV* Neovascular glaucoma, *LP* Light perception, *HM* Hand motion, *CF* Count finger

### IOP

**Fig. 1:**
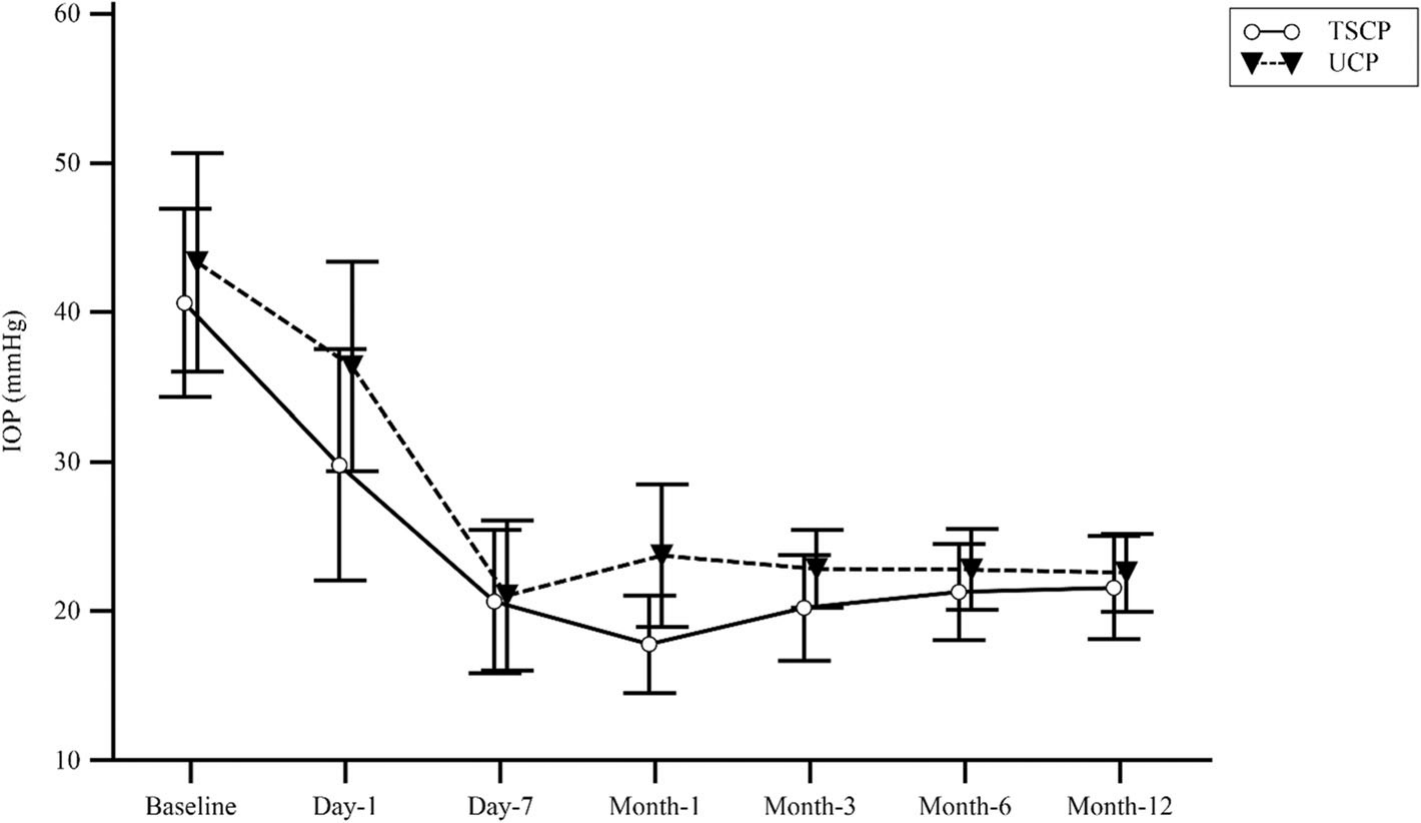
Comparison of changes in IOP before and after surgery between the two groups. Postoperative IOPs were significantly lowered in both procedures, compared with baseline. However, there is no difference in the trend of IOP reduction between the two groups

As shown in Fig. 1, the postoperative IOP was decreased in both groups, and the differences were statistically significant (*P*<0.01) compared with baseline. In UCP group, the 1-day, 7-day, 1-month, 3-month, 6-month and 12-month IOP values were 36.38 ± 12.15, 21.04 ± 8.74, 23.74 ± 8.27, 22.84 ± 4.55, 22.79 ± 4.68, and 22.57 ± 4.50 mmHg respectively, compared with 29.79 ± 13.43, 20.64 ± 8.32, 17.79 ± 5.59, 20.21 ± 6.15, 21.29 ± 5.61, and 21.57 ± 6.02 mmHg in TSCP group. There was no significant difference in the trend of IOP reduction between groups (*P* = 0.360). With the use of topical anti-glaucoma agents, the average IOP was 22.57 ± 4.50 mmHg in the UCP group 12 months after the operation, of which IOP in 9 eyes were less than 21 mmHg, accounting for 64.29% of eyes in that group. In contrast, the average IOP was 21.57 ± 6.02 mmHg in the TSCP group, of which 71.42% of eyes (*n* = 10 eyes) were less than 21 mmHg. Statistical significance was not reached for this difference. All patients experienced IOP reduction at month-12 among baseline IOP stratifications (Table 2).

**Table 2:** IOP value and the number of anti-glaucoma medication in each IOP stratification and treatment range at the final follow-up

Group	IOP Range	Treatment Range	n	Baseline IOP (mmHg)	12-month IOP (mmHg)	Baseline Medications	12-month Medications	Proportion of zero-agent (n)
**UCP**	21 < IOP ≤ 35	6 sections	5	28.28 ± 2.32	20.00 ± 5.39	2.40 ± 0.55	1.00 ± 1.00	40%(2)
	35 < IOP ≤ 45	8 sections	1	39.90	31.00	3.00	2.00	0
	IOP > 45	10 sections	8	53.23 ± 4.50	23.13 ± 2.42	2.13 ± 0.99	1.13 ± 0.83	25%(2)
**TSCP**	21 < IOP ≤ 35	20 shots	7	31.57 ± 2.51	19.57 ± 6.27	2.00 ± 0.82	1.00 ± 1.15	43%(3)
	35 < IOP ≤ 45	25 shots	2	40.00 ± 1.41	21.50 ± 0.71	3.00 ± 0.00	1.50 ± 0.71	0
	IOP > 45	30 shots	5	53.60 ± 5.18	24.40 ± 6.47	2.60 ± 0.55	1.80 ± 0.84	0

### Use of anti-glaucoma agents

**Fig. 2:**
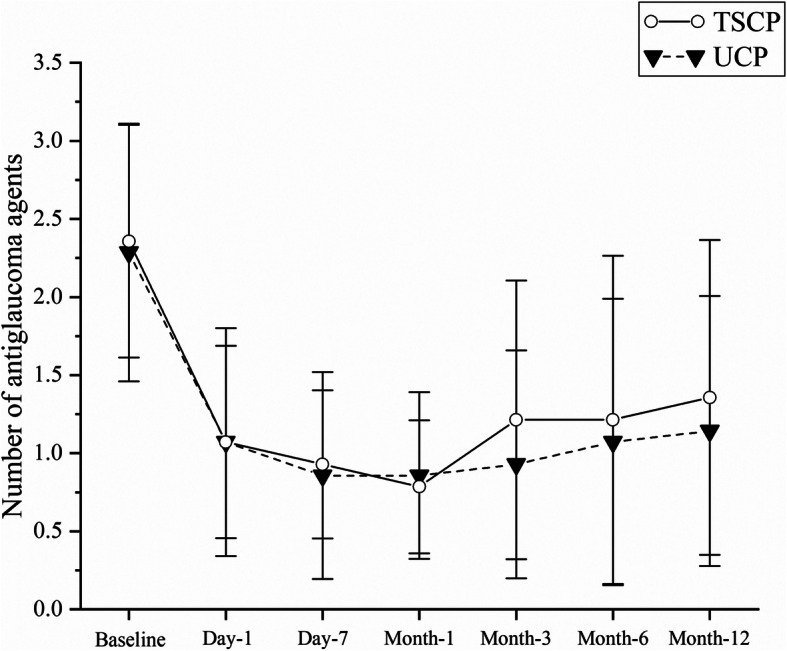
The number of anti-glaucoma agents used at baseline and each postoperative visit. In this diagram, the average number of anti-glaucoma medication was marked at each time point for each group. Compared with baseline, both groups showed a significant decrease in the number of medications, *P* < 0.01. No statistical difference was reached between groups

The number of anti-glaucoma agents used at baseline and after the procedure was shown in Fig. 2. At the last follow-up on month-12, the mean number of anti-glaucoma agents in the UCP group was decreased from 2.29 ± 0.83 before surgery to 1.14 ± 0.86, where the difference was statistically significant (t = − 5.551,*P* < 0.01), and that in the TSCP group was reduced from 2.36 ± 0.74 before surgery to 1.36 ± 1.01, where the difference was statistically significant (t = − 4.770, *P* < 0.01). However, the difference between the two groups was not statistically significant (F = 0.381, *P* = 0.540). As shown in Table 2, at month-12, all patients reduced their anti-glaucoma medications to a different extent with 4 patients free of medication in UCP group and 3 in TSCP group.

### Visual acuity

In the UCP group, the BCVA at the last follow-up was unchanged in all patients compared with that before surgery. In the TSCP group, the BCVA at the last follow-up was declined in 2 patients (14.3%) while unchanged in the remaining cases. There was no significant difference in the change of visual acuity between two procedures for the treatment of refractory glaucoma (Fisher’s exact probability method, *P* = 0.483).

### Postoperative complications and pain

As shown in Table 3, the pain score in the UCP group was significantly lower than that in the TSCP group within 1 day after surgery (Fisher’s exact test, *P* = 0.041). In terms of other complications, the total incidence of postoperative complications was 21.4% in the UCP group and was 64.4% in the TSCP group. However, the difference between the two groups did not reach statistical significance (**χ**^**2**^ = 3.646, *P* = 0.056). None of the patients had severe complications, such as eyeball atrophy, suprachoroidal hemorrhage and endophthalmitis.

**Table 3:** Postoperative pain score and complications

		UCP	TSCP	χ^**2**^value	***P-***value
**Pain grading**	**0**	9	2		0.041*
	**1**	3	6		
	**2**	2	4		
	**3**	0	2		
**Persistent conjunctival congestion**		1	4	0.974	0.324
**Transient corneal edema**		2	3	0.000	1.000
**Subconjunctival haemorrhage**		0	1		1.000*
**Scleral blot or conjunctival burn**		0	1		1.000*
**Sum**		3	9	3.646	0.056

*Fisher’s exact probability method

## Discussion

Refractory glaucoma represents a type of complex glaucoma that cannot be treated effectively using conventional filtering surgery and auxiliary anti-glaucoma agents. In the past, the standard therapeutic method was cyclocryotherapy. However, this approach exhibits considerable damage to the eye tissue and many complications and incurs significant pain to patients, which limits its application in clinical practice [8, 9]. Cyclodestructive surgery with LASER energy such as TSCP is a well-established alternative to cyclocryotherapy. UCP is a method that uses high intensity focused ultrasound (HIFU) to transmit power to the target organ, in which the ultrasound energy is converted into a thermal effect to coagulate the target organ without affecting the adjacent tissues [10, 11]. With the continuous improvement of the technology, UCP has come into the routine armamentarium of glaucoma surgeon. As in this study, we have shown that UCP had durable efficacy as laser coagulation in terms of IOP control in adult refractory glaucoma with less postoperative pain.

The entire UCP process takes only a few minutes. The operation is straightforward and incision-free, which results in low surgical risk and simple postoperative care [12, 13]. Preoperative evaluations include measuring the patient’s ocular axis, the white-to-white corneal diameter and the ciliary body size. These data may help the surgeons to select a proper probe. The number of treatment sectors should be determined based on the patient’s baseline IOP as mentioned. Relevant animal experiments have shown that it has a dual mechanism of reducing aqueous humour production and increasing aqueous humour outflow [14]. Besides, during UCP, the blood-aqueous water barrier is retained, which avoids the occurrence of severe inflammatory reactions. Histology has revealed that the thermal effects of ultrasound beams are sustainable and are safe for the retina [7, 15]. Earlier clinical studies on UCP have also shown its effectiveness and safety in treating glaucoma [16–18]. A recent study comparing UCP with cyclocryotherapy in eyes with neovascular glaucoma confirmed safety advantage of UCP and equivalent efficacy [19].

TSCP has also been widely used in clinical practice as an alternative to cyclocryotherapy, and it is no longer limited to the treatment of end-stage glaucoma. It causes heat damage to the ciliary body through the thermal effect, resulting in coagulative necrosis of the pigment epithelium, matrix and reduced blood supply to the ciliary body, thus reduces the secretion of aqueous humour by the ciliary process. In addition, the contraction of the ciliary body after photocoagulation also increases the drainage of aqueous humour to a certain extent. Furthermore, photocoagulation of the ciliary body crown can pull the iris root back to reduce the trabecular meshwork obstruction [20, 21]. The TSCP process is simple and is also incision-free, with low surgical risk and easy postoperative care [22]. Intraoperatively, the number and range of photocoagulation should be determined according to the baseline IOP of the patients and the surgeon’s experience. However, the suitable laser energy, the best number and range of photocoagulation points remain inconclusive around the world [23–25].

Results of the present study showed that both UCP and TSCP were effective in the treatment of refractory glaucoma. There were no significant differences in reducing IOP and the use of postoperative anti-glaucoma agents between the two groups. In terms of postoperative complications, UCP showed a lower pain score and a statistically insignificant but substantial trend of less mild complications. Both groups had no severe complications in our study, which echoed the safety of both modalities. Furthermore, two eyes were suffering declined visual acuity after TSCP compared with zero with UCP in our follow-up. These findings corroborate the idea of more accurate location and less nearby tissue injury with UCP procedure [26, 27].

Treatment range is a particularly important factor meriting considerations. M. Graber and colleagues reported their retrospective comparison of UCP with TSCP in refractory glaucoma in a French cohort, showing an inferior efficacy but fewer complications with UCP than with TSCP, which is slightly different from current results [28]. However, their treatment strategy was different from ours. For UCP, their treatment range was fixed to 6 sections, and for TSCP, they used higher pulse energy. In our practice, the strategy for both procedures should be personalized according to baseline IOP. In addition, a slight reduction of pulse energy in TSCP would prevent the eyes from severe complications. With the improvements in technology and techniques, UCP would achieve an efficacy equivalent to the traditional modalities.

The limitations of the present study include: (1) The sample size is relatively small with considerable heterogeneity, deriving from both glaucoma types and baseline IOP levels. However, we have balanced them between groups; (2) the follow-up time was short. All patients are still under follow-up. The long-term efficacy of the two procedures needs to be explored; and (3) as a retrospective study in nature, our results might be biased from the patient selection. Extrapolation should be cautious, and prospective studies should be done to confirm our findings.

## Conclusion

In summary, UCP and TSCP are both safe, effective and repeatable treatment methods for refractory glaucoma in China. The UCP is more straightforward in operation, easier to perform, and causing fewer complications than TSCP. However, its long-term efficacy in refractory glaucoma patients remains to be determined. Thus, it could be selected with considering patients’ preference and surgeons’ experience.

## Data Availability

The data used in the current study are available from the corresponding author upon request.

## Abbreviations

UCP: Ultrasound cycloplasty
TSCP: Transscleral cyclophotocoagulation
IOP: Intraocular pressure
BCVA: Best-corrected visual acuity
UBM: Ultrasound biomicroscopy
